# Assessing 2D visual encoding of 3D spatial connectivity

**DOI:** 10.3389/fbinf.2023.1232671

**Published:** 2024-01-22

**Authors:** Benedetta F. Baldi, Jenny Vuong, Seán I. O’Donoghue

**Affiliations:** ^1^ The Garvan Institute of Medical Research, Darlinghurst, NSW, Australia; ^2^ CSIRO Data61, Eveleigh, NSW, Australia; ^3^ School of Biotechnology and Biomolecular Sciences, University of New South Wales, Kennsington, NSW, Australia

**Keywords:** visual encoding, user study, visual analytics, spatial connectivity, chromatin organization, circular layout, adjacency matrix, half-matrix layout

## Abstract

**Introduction:** When visualizing complex data, the layout method chosen can greatly affect the ability to identify outliers, spot incorrect modeling assumptions, or recognize unexpected patterns. Additionally, visual layout can play a crucial role in communicating results to peers.

**Methods:** In this paper, we compared the effectiveness of three visual layouts—the adjacency matrix, a half-matrix layout, and a circular layout—for visualizing spatial connectivity data, e.g., contacts derived from chromatin conformation capture experiments. To assess these visual layouts, we conducted a study comprising 150 participants from Amazon’s Mechanical Turk, as well as a second expert study comprising 30 biomedical research scientists.

**Results:** The Mechanical Turk study found that the circular layout was the most accurate and intuitive, while the expert study found that the circular and half-matrix layouts were more accurate than the matrix layout.

**Discussion:** We concluded that the circular layout may be a good default choice for visualizing smaller datasets with relatively few spatial contacts, while, for larger datasets, the half- matrix layout may be a better choice. Our results also demonstrated how crowdsourcing methods could be used to determine which visual layouts are best for addressing specific data challenges in bioinformatics.

## 1 Introduction

Chromosome conformation capture (3C) techniques can give insight into how the three-dimensional (3D) organization of the genome influences gene transcription, and *vice versa* (Dekker et al., 2002; Dostie et al., 2006; Lieberman-Aiden et al., 2009; Li et al., 2010). These 3C techniques work by detecting chromatin fragments that are located close to each other in 3D space, resulting in a dataset of spatial contacts. These contact datasets are conceptually similar to the inter-atomic contact datasets inferred for proteins via nuclear magnetic resonance (NMR) spectroscopy in that they can provide information about spatial structure (Wrinch, 1965; Ernst et al., 1990; Rieping et al., 2005). However, unlike protein NMR spectroscopy, 3C techniques generally do not possess the resolution to reconstruct accurate or useful 3D models; as a result, the analysis of 3C data focuses on identifying patterns in the inferred spatial connectivities (Smallman et al., 2001; Plumlee and Ware, 2006). These patterns can, in turn, provide useful insights, e.g., by revealing which genes are influenced by a specific promoter (Acemel et al., 2017).

For identifying patterns in 3C data, Lieberman-Aiden et al. (2009) have proposed using adjacency matrices, and have used this visual layout method to develop JuiceBox, a visualization tool widely used for exploring 3C data (Durand et al., 2016).

The visual representation of macromolecular contact data using adjacency matrices (also known as distance matrices) has been common practice for many decades (Phillips, 1970). The matrix layout has been used to visualize various aspects of large macromolecules, such as structural domains (Kuntz, 1972) and solvent accessibility (Nishikaw and Ooi, 1974). Distance matrices have also been used as part of the process for deriving macromolecular structure from experimental data (Galaktionov and Rodionov, 1980), similarly to how 3C experiments are now being used.

**FIGURE 1:**
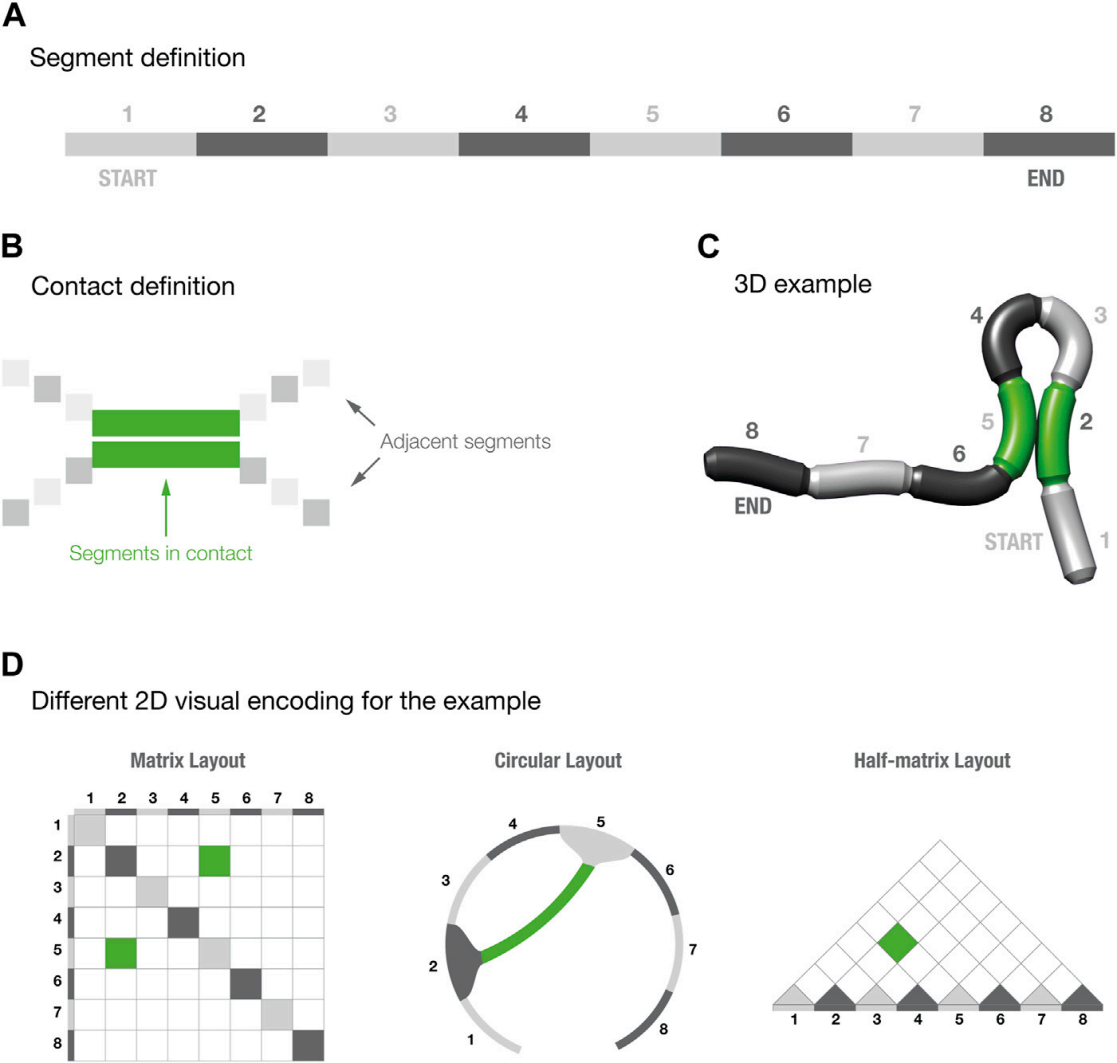
Segments and contact definition for three-dimensional models. Panels **(A–C)** provide a schematic explanation of how to read the three-dimensional models. Panel **(D)** shows the three different visual layouts for the 3D example shown in panel **(C)**.

However, spatial connectivity data can also be visualized with alternative layout methods. As mentioned before, a common alternative is the half-matrix (Figure 1). Contact matrices can often have the same sequence repeated on both sides of the diagonal, making them symmetrical. The half-matrix layout eliminates the symmetry and data redundancy by removing half of the matrix. This layout is used for the visual exploration of 3C data in the widely used WashU Epigenome Browser (Zhou et al., 2011).

Another alternative method to visualize contact data is via a circular layout (Figure 1); such layouts are commonly used to show spatial contacts in RNA structures (Hajdin et al., 2013), but are less commonly used for 3C data. Several tools are available to generate circular layouts, of which one of the more widely used is Circos—a tool initially designed for comparative genomics and cancer datasets (Krzywinski et al., 2009).

The choice of visual layout method can be crucial, especially with data that are complex, high-dimensional, and have variable uncertainty (Gehlenborg et al., 2010; Pavlopoulos et al., 2015; O’Donoghue et al., 2018). For assessing the suitability of such layouts, the term *visual effectiveness* is often used to describe the ability of a particular visual layout to exploit the capabilities of the output medium and of human visual perception to enable data to be accurately read, or visually decoded, by a viewer (Mackinlay, 1986; O’Donoghue et al., 2018). Another related term is *visual expressiveness*, which describes how well a visual encoding expresses the information most relevant to the phenomena studied, and how quickly a viewer can visually decode the information (Dastani, 2002; O’Donoghue et al., 2018). Using effective and expressive visual layouts can greatly affect the ability to identify outliers, spot incorrect modeling assumptions, or recognize unexpected patterns that might otherwise elude automated analysis approaches (O’Donoghue et al., 2018). Additionally, visual layout can play a crucial role in communicating results to peers. These considerations apply for all scientific data, but are especially relevant for exploratory analysis in emerging fields, such as 3C techniques, where there are many unknowns.

Cleveland and McGill (1984, 1986) pioneered the use of perceptual studies to assess the effectiveness and expressiveness of visual layouts, via a straightforward but laborious method. They introduced the concept of elementary perceptual tasks, in which a user is presented with a range of visual layouts and asked to infer basic properties, and their responses are then assessed for accuracy. Typically, these perceptual tasks involve estimating quantitative values that have been encoded using different visual channels, such position on a common scale, length, area, volume, direction, or shading. Perceptual studies such as these can assess the accuracy, precision, and speed that people achieve when using a specific visual encoding strategy.

Several perceptual studies mentioned above have concluded that the adjacency matrix is a powerful and broadly useful visual layout compared with graph-based layouts, in which connections are drawn explicitly (Munzner, 2014). Most notably, because connections are omitted, matrix layouts can show larger and more complex datasets clearly, especially when used with reordering methods (Behrisch et al., 2016). On the other hand, matrix layouts also have well-known limitations, especially when used to encode quantitative data, for example, in a heat map, where optical illusions can lead to surprising decoding errors (Wong, 2010). In addition, there are specific use cases where matrix layouts are considered less intuitive than tailored, graph-based layouts. For example, phylogenetic relationships are commonly visualized using tree graphs (Procter et al., 2010).

Previous studies have indicated that graph-based layouts (a broad category that includes the circular layouts used for 3C data) can be better than matrix layouts for specific tasks, such as *path finding*, i.e., recognizing connections between nodes, when the data or the density of the connection is small (Ghoniem et al., 2005; Behrisch et al., 2016). Conversely, when data density increases, a node-link graph can become a *hairball*, in which individual nodes or edges cannot be visually resolved. In such cases, the matrix appears to be a more effective visual layout (Ghoniem et al., 2005; Behrisch et al., 2016).

In this work, we focused on the specific case of visualizing spatial connectivity derived from simple 3D objects (e.g., a simplified schematic of a macromolecule), and we hypothesized that, for this case, matrices may be less suitable than connectivity graphs based on circular layouts. Circular layouts are arguably more intuitive to interpret, since spatial connectivity is explicitly encoded by a connecting line or arc, rather than needing to be inferred based on location. Thus, circular layouts are more expressive, as they provide a more direct correspondence between connectivity data and how the visual channel used to encode connectivity is perceived (Dastani, 2002).

To understand which visual layout is better suited to represent spatial connectivity data, we set up two studies inspired by the work of Cleveland and McGill (1984, 1986), comparing the accuracy of the matrix, half-matrix and circular layouts (Figure 1). Both studies used Versus (Vuong et al., 2018), a framework developed in-house to streamline perceptual studies. One study recruited participants via an online crowdsourcing platform, using the approach pioneered by Heer and Bostock (2010). A second “expert” study recruited participants actively working as biomedical research scientists.

## 2 Materials and methods

In planning the studies for this paper, we first did rough estimates of the likely budget and time requirements for deriving statistically meaningful results. Based on these estimations, we decided that each of our studies would ask 15 unique multiple-choice questions, each with five possible answers, and each repeated once, giving a final total of 30 questions per layout. We ran two studies: one comprising laypeople recruited online and a second “expert” study comprising scientists recruited from three biomedical research institutions. Both studies were conducted using Versus (Vuong et al., 2018), a web-based tool developed in-house to aid in creating and running multiple-choice perceptual surveys.

One of our key goals in the design of these studies was to answer the question “Which one of these three layouts better encodes three-dimensional connectivity?”. To address this, we decided to use a low level of complexity in both the 3D model and the three visual layouts, thereby minimizing the confounding effects that often occur with large and complex datasets. As a result, our study focused on determining which layout expresses spatial connectivity data in a way that can be most effectively read by the study participants.

### 2.1 Three-dimensional models

We created a set of segmented, cylindrical 3D models using the open source 3D modelling software Blender (Hess, 2010). To keep complexity to a minimum while allowing for a sufficient number of possible contacts, we fragmented the cylinder into eight segments (Figure 1A). The eight segments were then initially colored using two alternate shades of gray, beginning with a light gray coloring in the first segment. Since there were an even number of segments, the first and last segments were initially colored light and dark gray, respectively, thus giving each model a visually distinct directionality.

We decided to display only one contact per model to minimize the effects of misreadings. We then created 15 different models with different conformations, that resulted in different pairs of segments forming a contact. To increase the readability of the 3D structures, we highlighted the contact-forming segments by changing their color to green (Figures 1B, C). To increase the sense of depth in the models, and therefore improving their readability, we created animated images by using a standard rocking motion about the *y*-axis with an amplitude of 3° and comprising 136 frames per cycle, displayed over 3 s (Brooks et al., 2014).

### 2.2 Visual layouts

For each model, we then created a circular, half-matrix, and matrix layout that provided a 2D visual encoding of the spatial contact in the model. We designed these visual layouts to maximize clarity and consistency with the corresponding 3D models. Firstly, each layout utilized the same color scheme as the 3D models. The same alternate shades of gray were used for all layouts to indicate the segment numbers, and the same green coloring was used to indicate contacting segments. Secondly, we decided to not take into account intrasegment contacts. In the matrix layout, this resulted in coloring all the diagonals with the same alternate shades of gray, representing the segment positions (Figure 1D). For the half-matrix layout, however, it translated into coloring the base of each visualization with the same shades of gray.

### 2.3 Incorrect answers

Since the survey comprised a multiple-choice questionnaire, we had the possibility of introducing targeted incorrect answers. Our aim was to have an incorrect answer that could distinguish cases in which the participant understood the 2D visual layout but misread the 3D model. Thus, we introduced, whenever possible, what we call an *inverted answer*, i.e., an answer that would be correct if the model was read in the opposite direction. These inverted answers let us identify cases where a participant mistakenly switches the first and last segments. Of the 15 models we generated, three did not have an inverted answer, due to the symmetric position of the spatial contact; a full list of the cases and their inverted counterparts is provided in Table 1.

**TABLE 1:** Inverted answers for each type of three-dimensional contact.

Contact	Inverted contact
1–4	5–8
2–5	4–7
3–6	*none*
4–7	2–5
5–8	1–4
1–5	4–8
2–6	3–7
3–7	2–6
4–8	1–5
1–6	3–8
2–7	*none*
3–8	1–6
1–7	2–8
2–8	1–7
1–8	*none*

### 2.4 MTurk study

Recruiting participants for perceptual studies can be time-consuming and expensive. Launched in late 2005 as a crowdsourcing internet marketplace, Amazon’s Mechanical Turk (MTurk) provides a way to make this process quicker and less expensive. Most (88%) MTurk workers are under 50 years of age and many (51%) have a college degree; otherwise, they have highly variable demographics (Sheehan, 2018).

Heer and Bostock (2010) were the first to utilize MTurk for perceptual studies, and show that it could be used to reproduce the results obtained by Cleveland and McGill (1984; Cleveland and McGill, 1986). Since then, many other perceptual studies have been carried out using crowdsourcing platforms, although such studies are still uncommon in bioinformatics (Mason and Suri, 2012; Klippel et al., 2015; Borgo et al., 2018). For these reasons, we decided to utilize MTurk to recruit 50 participants for each layout, resulting in a final total of 150 people and 4,500 data points.

#### 2.4.1 Qualification

From the MTurk qualification criteria, we selected workers that had an Amazon MTurk approval rate greater than 85%. Workers were paid US$ 4 for each “HIT” (“Human Intelligence Task”) they performed with MTurk. This payment was set to be at least equal to the average USA federal minimum wage of US$ 11.5/h.

Before participating in the study, each worker was required to: 1) digitally sign a consent form (Supplementary Figure S1); 2) study an explanatory paragraph that explained how to read the 3D models (Supplementary Figure S2); and 3) take and pass a qualification test. We devised this qualification test to assess if the participants understood the 3D models, thereby allowing us to select only workers that could provide insightful results. The qualification test comprised four multiple-choice questions on how to read the 3D models. No questions about the three visual layout were introduced at this stage. We chose four out of the 15 models to be used in the qualification test, and new animated images were created using different orientations to the ones used in the study itself, with the goal of minimizing learning effects for these models.

For each qualification question, we asked which segments formed the contact, and provided five possible answers: a correct answer, three random incorrect answers, and the inverted answer. For two of the models, we clearly labelled the contacting segments in the animated images, while for the other two, these labels were removed.

This qualification test was designed to screen out MTurk workers that provided responses indicating they were unable or unwilling to read the 3D models. Only workers who correctly answered all qualification questions were allowed to participate further in the study. To arrive at our desired final total of 150 qualified MTurk participants, we ended up running this qualification test on 251 MTurk workers, of which 101 were disqualified based on their test responses.

### 2.5 Expert study recruitment

We recruited 10 experts for each visual layout, making a total of 30 expert participants and 900 data points. Recruitment was performed via email to employees of the Garvan Institute of Medical Research, St. Vincent Hospital, the Kinghorn Cancer Centre and the Victor Chang Cardiac Research Institute. Our email asked for the participation of PhD students, postdoctoral researchers, and principal investigators. We were able to verify that the resulting cohort were all scientists actively working in biomedical research, so all of them had broad familiarity with creating and interpreting figures in biomedical publications. Thus, we described this as the expert cohort, to distinguish them from the MTurk participants. Note that the expert cohort was not selected based on their familiarity with 3C data, adjacency matrices, or circular layouts.

The expert participants, after giving their consent, were given the same explanatory paragraph as the MTurk workers on how to read the 3D models. After that, they immediately started the study without going through the qualification test. To protect the privacy of participants, we have not provided individualized demographic information about each participant. As thanks to the participants, we offered a coffee voucher at a local cafeteria.

### 2.6 Visual analytics via versus

Versus is a web-based tool that facilitates the creation of image comparison studies based on either two-alternative forced-choice (2AFC) or multiple-choice questions (Vuong et al., 2018). Versus can be used as a standalone tool or in conjunction with MTurk to quickly and conveniently recruit many study participants. For this work, we used the multiple-choice module and the integration with MTurk for the MTurk study and the standalone version for the expert study. The Versus algorithm randomizes the order in which the questions are presented to each participant, and also the order of the answers in each question. This randomization is essential, as our design study required the use of duplicated questions for consistency and quality control. The number of question to be repeated can be set using the *fraction to be repeated* parameter. All the input files used in this study were deposited as a published dataset on the Open Science Framework (Baldi, 2018). An example of the multiple-choice question presented to the participants can be seen in Supplementary Figure S3.

### 2.7 Analysis of results

The analysis of the results was carried out in R (R Core Team, 2018) using two separate R scripts, both of which were included in the Open Science Framework dataset (Baldi, 2018). The *p*-values were adjusted for multiple testing using a standard method (Benjamini and Hochberg, 1995), with *α* set to 0.05.

## 3 Results

### 3.1 MTurk study

Our first step in assessing the results of the MTurk study was to analyze the consistency of each participant’s response. This analysis was based on the replicated questions included in each questionnaire (Meade and Craig, 2012; Cheung et al., 2017). By analyzing the response to these replication questions, we could derive a consistency score for each participant, then we could see how consistency varied across the three visual layouts.

**FIGURE 2:**
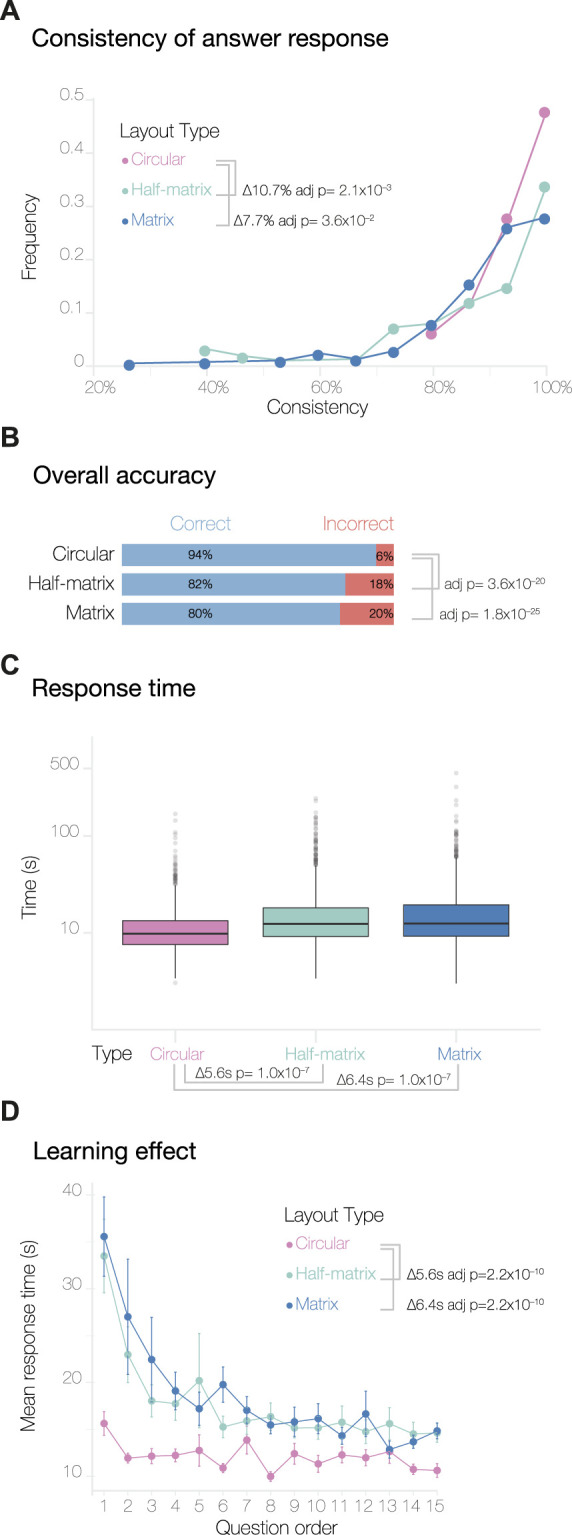
Overview of results from the MTurk study. Panel **(A)** shows the consistency levels calculated by comparing the answer responses and their duplicates. The overall accuracy of the answers and the response time, plotted on a log scale, are shown in panels **(B,C)** respectively. Panel **(D)** shows the learning effect curves, calculated by averaging the response times for duplicate questions (see Methods).

We found that the consistency score varied significantly across the three layouts (Figure 2A). The half-matrix layout shows a decrease in the overall consistency score of 10.7% (adjusted *p*-value = 1.2 × 10^−3^) compared to the circular layout, and the matrix shows a decrease of 7.7% (adjusted *p*-value = 3.6 × 10^−2^). Here, the *p*-values were determined by an analysis of variance (one-way ANOVA) with Tukey’s method used for *post hoc* analysis. We discarded responses from participants that scored ≤20% consistency, which is the threshold for complete randomness for this multiple-choice study design.

#### 3.1.1 Accuracy

Next, we analyzed participant accuracy across the three visual layouts. Overall, the level of accuracy was high; this was expected, since we designed the test data to be minimal and relatively easy to understand. To assess if the layouts had an impact on the participant’s accuracy, we performed a Pearson’s Chi-squared test of independence with the null hypothesis that participants can decode the information equally well from any of the three layouts. Thus, our alternative hypothesis was that participants decode the information displayed in the three layouts differently. Figure 2B shows that participants achieved significantly higher accuracy using the circular layout compared to the half-matrix and the matrix layout (adjusted *p*-value = 3.6 × 10^−20^ and 1.8 × 10^−25^, respectively, with a pairwise test for independence for the nominal data). There was no statistically significant difference between the overall accuracy of the matrix and half-matrix layouts.

#### 3.1.2 Intuitiveness

We then compared the expressiveness or intuitiveness of the three visual layouts. Since we did not train the participants to read any of the visual layouts (only the 3D models), we assumed that the time required to answer a question could be used as a measure of how intuitive a visual layout was to the participant. From the 4,500 responses, we removed one single response for this analysis, as it was an outlier with a >5000s response time, which is likely explained by the participant being interrupted during the survey. We did an ANOVA analysis comparing the remaining response times across the three visual layouts (Figure 2C). This showed that the response time for the circular layout was significantly shorter (by 6.4*s*) than for the matrix layout (Tukey *post hoc* adjusted *p*-value = 1.0 × 10^−7^), and also significantly shorter (by 5.6*s*) than for the half-matrix layout (Tukey *post hoc* adjusted *p*-value = 1.0 × 10^−7^).

We also calculated a second measure of intuitiveness derived from learning effect curves. As each participant progressed through the survey, they typically learned to carry out subsequent tasks more efficiently, with the result that the time to complete each task decreased until a plateau was reached (Figure 2D). The pattern was seen for all visual layouts, however for the circular layout the plateau was reached significantly more rapidly, as assessed by an ANOVA followed by Tukey *post hoc* analysis (*p*-values are given in Figure 2D). We encountered a slight complication in generating and analyzing these learning effect curves, due to the fact that the version of Versus we used did not record the order in which duplicated questions were shown. We addressed this by averaging all the duplicate responses with their respective counterparts. As a result, the learning curves obtained show response times for questions 1 through 15, rather than 1 through 30.

#### 3.1.3 Inversion errors

Next, we assessed how many of the incorrect answers could be attributed to a simple inversion in counting segments in the 3D models. This type of error is not linked to how people read the data presented in the 2D visual layouts, but is instead linked to how the participants read the 3D models. This analysis was possible thanks to the incorporation of an inverted answer, as explained in the study design. For the circular layout, 84.5% of the incorrect answers can be attributed to inversion errors. For the half-matrix and matrix, the inversion error accounts for 56.6% and the 60.5% of the total errors, respectively (Figure 4A). Performing a Chi-squared test of independence, we saw a statistically significant difference between the percentage of inversion error in the circular layout compared to the matrix and the half-matrix layouts, with no significant difference between the latter two layouts.

#### 3.1.4 Inter-segment distance errors

**FIGURE 3:**
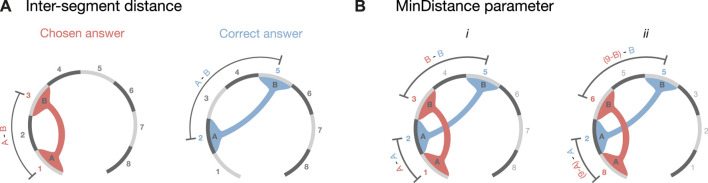
Error parameters definitions. As an example, we considered a possible set of answers for the 3D model presented in Figure 1C. The correct answer colored in light blue shows the contact between the segments 2 and 5, while the hypothetically chosen answer shows the connection between segment 1 and segment 3. **(A)** Shows how the inter-segment distance parameter was calculated (Eq. 1). **(B)** shows how the *MinDistance* parameter was calculated (Eq. 2). *i* depicts the first term, in which we calculate the distance between the first segment of the chosen answer minus the correct one added to the distance between the second segment of the chosen minus the correct answer. *ii* depicts the second term of Eq. 2 calculated as in *i* but inverting the directionality of the segments (e.g., segment 1 becomes segment 8).

We then assessed how many incorrect answers were caused by misjudging inter-segment distances (Figure 3A), versus how many were caused by miscounting the segment numbering, but still had the correct inter-segment distance. For this assessment, we defined the following inter-segment distance score between segment A (*Seg*(*A*)) and B (*Seg*(*B*)) as:

$$\begin{array}{c} |Seg\left(A\right)-Seg\left(B\right){|}_{\mathrm{Chosen}}-|Seg\left(A\right)-Seg\left(B\right){|}_{\mathrm{Correct}} \end{array}$$ (1)

This score can have the following values:

• *Inter-segment Distance* = 0 indicates incorrect answers that nonetheless had the correct inter-segment distance; inversion errors fell into this category.
• *Inter-segment Distance* >0 indicates answers where the inter-segment distance was too large.
• *Inter-segment Distance* < 0 indicates answers where the inter-segment distance was too small.

**FIGURE 4:**
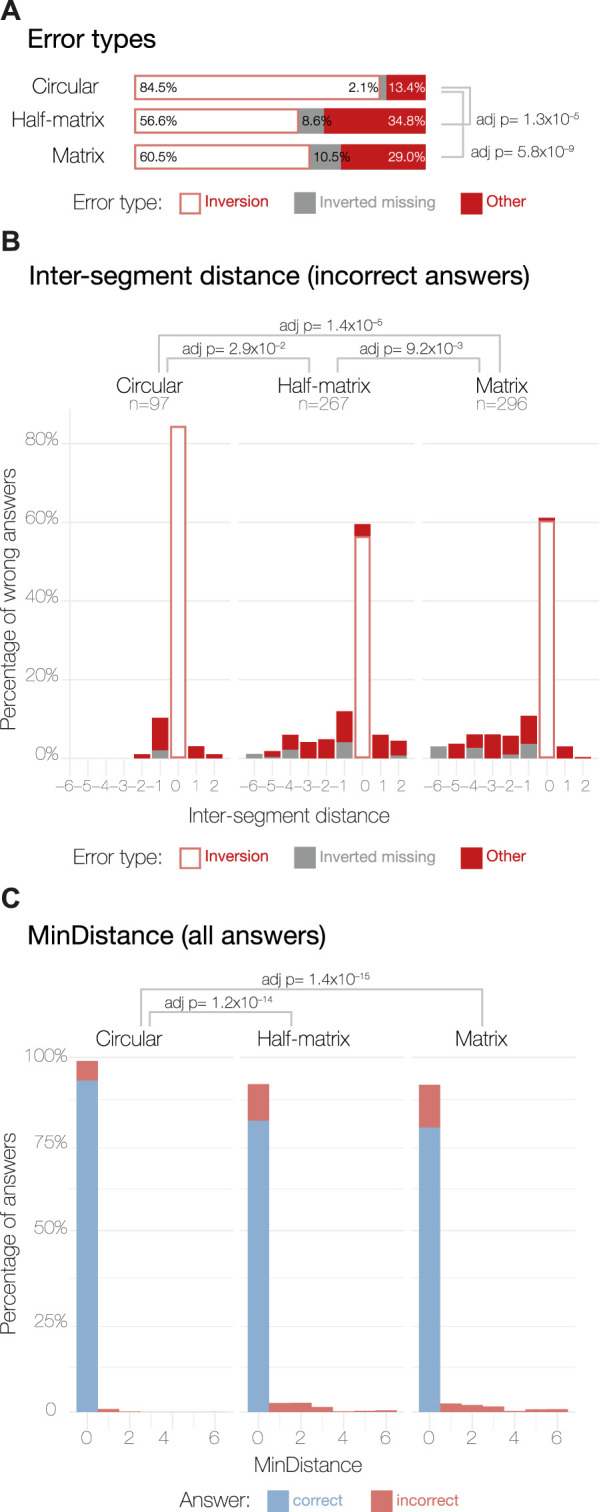
Error analysis for MTurk study results. Panel **(A)** shows the three possible errors types: (1) *inversion errors* happen when a participant selects the correct inter-segment distance, but switches the segment numbering; (2) *inverted missing* indicates a case where inversion errors were excluded by symmetry (Table 1); (3) *other* indicates all remaining errors. Panel **(B)** shows the distribution of the inter-segment distance parameter calculated as described in Eq. 1 for the incorrect answers only. The incorrect answer sample sizes are displayed under their respective layout labels. Panel **(C)** shows the distribution of the *MinDistance* parameter across all the answers, calculated as in Eq. 2.

Comparing inter-segment distance errors across the three visual layouts (Figure 4B), we found significantly different distributions, as assessed by pairwise Kruskal–Wallis non-parametric tests for independence and by Dunn tests for the *post hoc* analysis (Zar et al., 2010).

Overall, the circular layout shows a normal-like distribution with small deviations around 0; having −1 as the most common non-zero value, but positive values of 1 and 2 are also present in the distribution. By contrast, the half-matrix and matrix layouts both resulted in errors skewed towards a negative inter-segment distance score; i.e., participants using these layouts often chose contacts that were formed by segments in closer proximity than the correct answer.

The discretized nature of the contact data made it challenging to precisely quantify how much an answer deviated from its true value (Simkin and Hastie, 1987). To circumvent this issue, we calculated a new parameter that takes into account the relative position of the segments, without penalizing for inversion errors, which are not linked to layout errors (Figure 3B). This parameter, called *MinDistance*, was calculated across all the answers as:

$$\begin{array}{cc} \min\left(\right.\\ \left[Seg{\left(A\right)}_{\mathrm{Chosen}}-Seg{\left(A\right)}_{\mathrm{Correct}}+Seg{\left(B\right)}_{\mathrm{Chosen}}-Seg{\left(B\right)}_{\mathrm{Correct}}\right],\\ & \;\left[Seg{\left(\left|9-A\right|\right)}_{\mathrm{Chosen}}-Seg{\left(A\right)}_{\mathrm{Correct}}+Seg{\left(\left|9-B\right|\right)}_{\mathrm{Chosen}}-Seg{\left(B\right)}_{\mathrm{Correct}}\right]\\ & \left.\;\right) \end{array}$$ (2)

In this equation, the second term was calculated as the segment for the chosen answer, if it was inverted (e.g., segment 1 becomes segment 8), achieved by subtracting 9 from the segment number. Similar to the inter-segment distance score, the *MinDistance* parameter is equal to 0 for inverted errors and is directly proportional to the discrepancy in position between the chosen segments and the correct one. Therefore, the smaller the *MinDistance*, the more accurate is the answer. The *MinDistance* parameter was calculated across all the answers, resulting in a score that measures the overall error.

As for the inter-segment distance, we tested for differences in the distribution of the *MinDistance* parameter across the three layouts using the Kruskal–Wallis non-parametric test of independence. We found significant differences between the circular layout and the other two layouts (Figure 4C), with the circular layout resulting in more correct answers. However, we found no significant differences between the matrix and half-matrix layouts (Figure 4C).

### 3.2 Expert study

**FIGURE 5:**
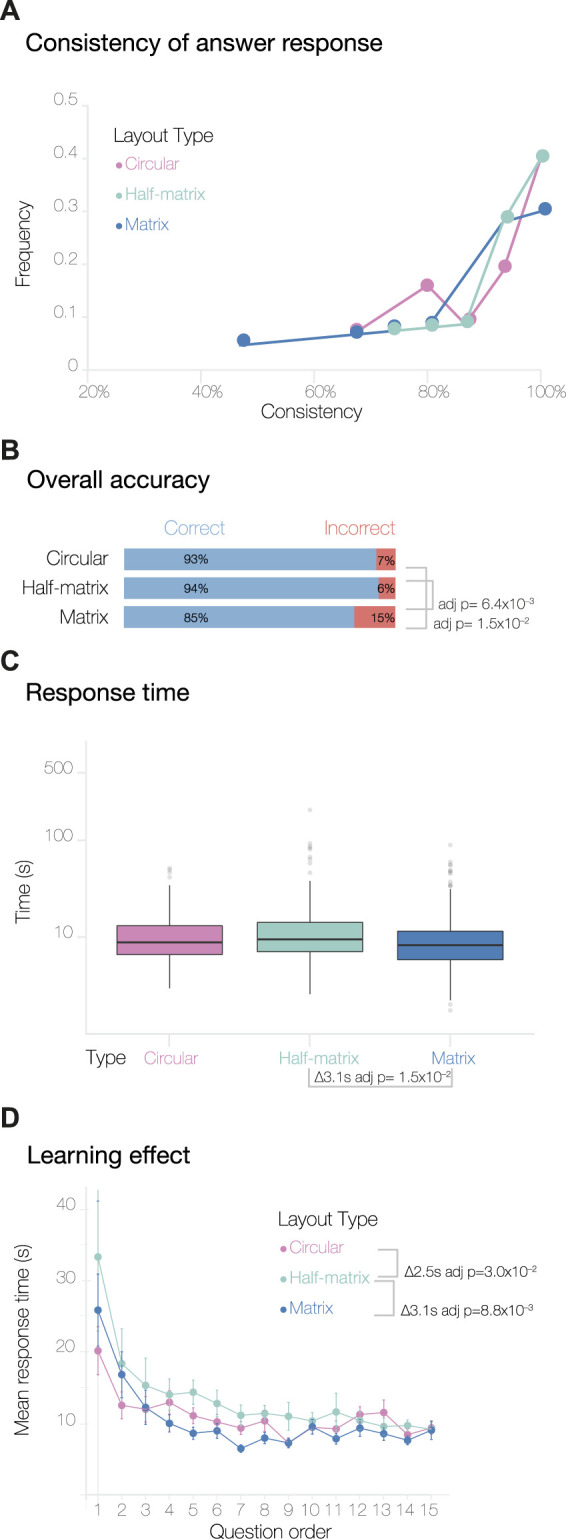
Overview of results from the expert study. **(A)** Shows response consistency to duplicated questions. **(B)** Show overall accuracy of the answers. **(C)** Shows response times plotted on a log scale. **(D)** Shows learning effect curves, based on participant response times to consecutive questions.

We analyzed the expert study data with the same methods we used for the MTurk study data. In contrast to MTurk participants, the consistency of answers in the expert study did not appear to vary with visual layout (Figure 5A). This may be accounted for by the smaller number of expert participants.

The overall accuracy of the answers was also quite high, as expected for participants more accustomed to reading similar visual layouts (Figure 5B). The accuracy obtained using circular and half-matrix layouts were not significantly different, while the accuracy obtained with the matrix layout was significantly lower (Figure 5B).

Interesting, although using the matrix layout resulted in the lowest accuracy, it also resulted in the shortest average response time (Figure 5C). Likewise, when looking at the learning curves, the matrix layout presented a similar profile to the circular layout, while the half-matrix showed significantly longer responses (Figure 5D). The learning curves showed less variability compared to the MTurk study, where the circular layout was clearly separated from the other two. This supports the idea that some visual layouts require more training than others, and that prior knowledge may have reduced this variability.

**FIGURE 6:**
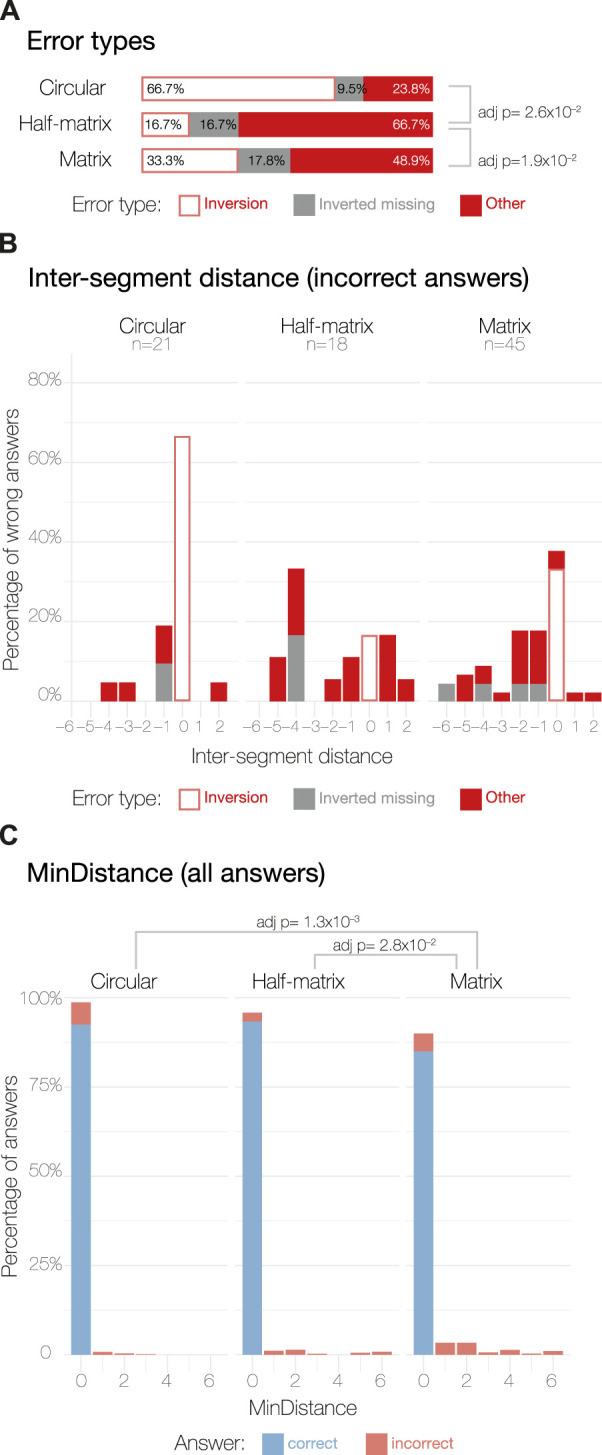
Error analysis for expert study results. **(A)** Shows the distribution of errors due to inversion, errors seen when no inversion was present, and all other errors. **(B)** Shows the distribution of inter-segment distances seen in incorrect answers. **(C)** Shows the distribution of MinDistance values across all the answers.

By analyzing errors in the expert responses, we saw that, compared to the circular layout, both matrix and half-matrix layouts had a significantly higher fraction of errors classified as *inverted missing* and *other* (Figure 6A). This implies that the percentage of errors that could be attributed to misreading the 3D model was lower for the matrix and half-matrix layouts when compared to the circular layout (Figure 6A).

We also saw that erroneous responses from experts were skewed towards negative inter-segment distances scores (Figure 6B), indicating a tendency to misassign the spatial contacts to segment pairs that were too close together in sequence (Figure 1A). As seen with the MTurk results, the circular layout resulted in the highest percentage of inversion errors (Figure 6B), indicating that this layout appeared to be better at conveying inter-segment distance.

Looking at the distribution of *MinDistance* scores, we found that the matrix layout was significantly less accurate than the circular and half-matrix layouts, while we found no statistically significant difference between the circular and the half-matrix layouts (Figure 6C).

## 4 Discussion

In this paper, we assessed the effectiveness of three visual layouts by measuring accuracy—i.e., the overall percentage of correct answers for each layout—as well as determining the incidence of errors unrelated to misreading the 3D models, based on inter-segment distance scores and the *MinDistance* parameters. For each layout, we also assessed visual expressiveness, or intuitiveness, by measuring average response times and learning curve effects.

For the MTurk study (Figure 2), we found that the circular layout gave the highest accuracy. Not only did this layout have a higher percentage of correct answers and error parameters close to zero, but the majority of errors that participants made appeared to have resulted from misreading the 3D model, not from misreading the 2D circular layout. In addition, the circular layout showed the shortest response time and an almost flat learning curve, indicating that this layout was more intuitive than the matrix and half-matrix layouts.

Interestingly, in the MTurk study, we observed unexpected behavior in the consistency of responses to the duplicated questions: both the half-matrix and matrix layout led to significantly lower consistency compared to the circular layout (Figure 2A). Taken together, the above results indicate that matrix-based layouts might not be the best visual encoding of 3D spatial connectivity data for non-expert audiences.

As reported in the introduction, adjacency matrices are widely used by the scientific community. While previous perceptual studies have found that this matrix layout can sometimes lead to significant errors, the authors of these studies have speculated that these errors may reduce once participants are trained to read the matrix layout (Dastani, 2002; Ghoniem et al., 2005; Munzner, 2014). For this reason, we were interested to recruit participants already familiar with reading scientific figures. As expected, expert participants showed greater confidence than laypeople in reading the 3D models and three visual layouts (Figures 2, 5). In addition, experts had similar learning curves with all three layouts (Figure 5D), although the half-matrix layout appeared to be slightly less intuitive than the matrix or circular layouts.

Similar to the MTurk study, the expert study found that using the circular layout resulted in the lowest error rate (Figure 5B) and in fewer egregious errors (Figure 6A).

Interestingly, our results suggested that errors unrelated to misreading the 3D model were higher for experts (Figure 6A) than for MTurk participants (Figure 4A). Possible explanations for this include: 1) the experts may have been overconfident, 2) the MTurk participants may have been more motivated, since they were paid for their responses; or 3) we did not require the experts to pass the qualification test used to screen MTurk participants. Further investigation into these explanations could be a useful goal for future studies.

Another interesting observation that may warrant further investigation was that errors in inferring the inter-segment distance were skewed to negative values—this effect was seen in both the MTurk (Figure 4A) and expert studies (Figure 6A), and across all three layouts, although the effect was somewhat less for the circular layout.

While the above results indicated that the circular layout had advantages over matrix-based layouts, the applicability of these results is limited by the very simple datasets used in our study design. For more complex datasets, previous perceptual studies suggest that matrix-based layouts are likely to be more effective, as mentioned in the Introduction (Ghoniem et al., 2005; Behrisch et al., 2016). Follow-up studies could be useful for exploring the data complexity thresholds at which a matrix-based layout becomes more effective than a circular layout. It would also be interesting to explore if the advantages of circular layouts can be extended to more complex datasets using data reduction techniques, such edge-bundling (Holten and Van Wijk, 2009). Additionally, it may be useful to determine if the advantages of both visual layouts could be combined by using each to generate two simultaneous views of the same dataset, each connected by interactive brushing and linking (Bremm et al., 2011).

Another interesting direction for future perceptual studies could be to assess different strategies of visually encoding contact strength, which is often a critically important variable, especially for spatial contacts derived from 3C techniques. In a matrix-based layout, contact strength is often encoded as color saturation or brightness, resulting in a heat map, while in the circular layout, it is often encoded as arc thickness. Based on previous studies, we would expect arc thickness to be a more effective visual encoding (Cleveland and McGill, 1984; Cleveland and McGill, 1986; Munzner, 2014); however, those studies were conducted in markedly different contexts. Thus, it would be useful to verify whether these expectations apply to spatial contact datasets.

It would also be interesting to explore the applicability of these perceptual studies in evaluating other common tasks involved in interpreting 3C data, such as visually comparing connectivity across multiple datasets (Ballweg et al., 2018).

## 5 Conclusion

In summary, our study indicated that the circular layout may be a good, default choice for visualizing small datasets with relatively few spatial contacts. For larger datasets, the half-matrix or matrix layouts may be a better choice. Further studies with larger sample sizes would be need to test the generality of these conclusions and to establish thresholds for switching between these two layouts.

Our study also demonstrated how emerging crowdsourcing methods can be used to determine which visual layouts are best for addressing specific data challenges in bioinformatics. Given the increased simplicity, affordability, and speed of such crowdsourcing methods, we would argue for wider use of these methods in the bioinformatics community.

## Data Availability

The datasets presented in this study can be found in online repositories. The names of the repository/repositories and accession number(s) can be found in the article/Supplementary Material.
