# Supplementary material for: Assessing 2D visual encoding of 3D spatial connectivity
Source: Front Bioinform. 2024 Jan 22;3:1232671. doi: 10.3389/fbinf.2023.1232671 (PMC10845337; doi:10.3389/fbinf.2023.1232671)
Supplement: Supplementary file 1 [file DataSheet1.pdf]

Supplementary Material

1 SUPPLEMENTARY DATA

The input files, user responses, and analysis files used in this study have been deposited on the Open Science Framework website at `https://osf.io/qxcn5`.

2 SUPPLEMENTARY FIGURES

2.1 Figures

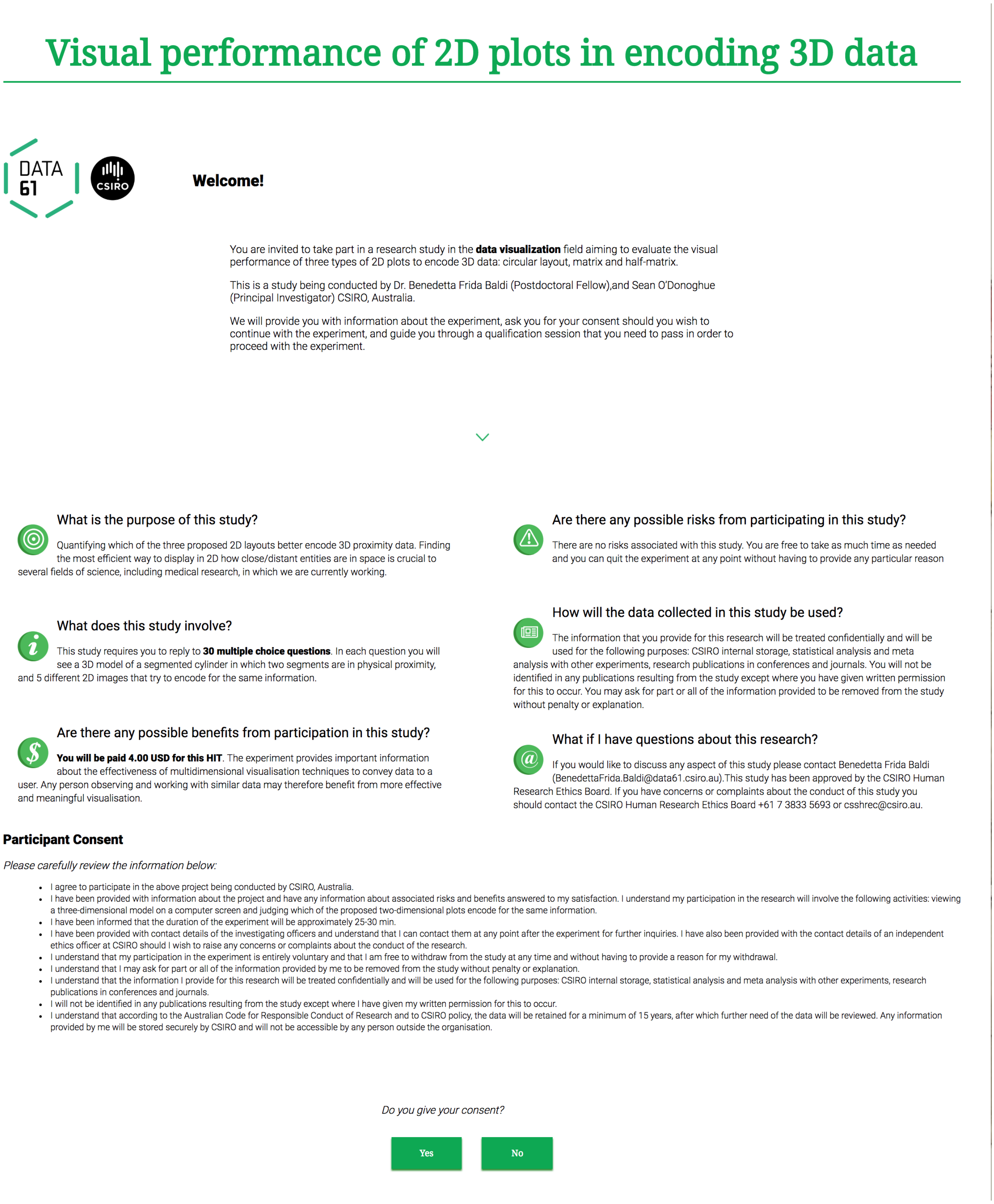

Visual performance of 2D plots in encoding 3D data

DATA 61 CSIRO

Welcome!

You are invited to take part in a research study in the **data visualization** field aiming to evaluate the visual performance of three types of 2D plots to encode 3D data: circular layout, matrix and half-matrix.

This is a study being conducted by Dr. Benedetta Frida Baldi (Postdoctoral Fellow),and Sean O'Donoghue (Principal Investigator) CSIRO, Australia.

We will provide you with information about the experiment, ask you for your consent should you wish to continue with the experiment, and guide you through a qualification session that you need to pass in order to proceed with the experiment.

What is the purpose of this study?

Quantifying which of the three proposed 2D layouts better encode 3D proximity data. Finding the most efficient way to display in 2D how close/distant entities are in space is crucial to several fields of science, including medical research, in which we are currently working.

What does this study involve?

This study requires you to reply to **30 multiple choice questions**. In each question you will see a 3D model of a segmented cylinder in which two segments are in physical proximity, and 5 different 2D images that try to encode for the same information.

Are there any possible benefits from participation in this study?

You will be paid 4.00 USD for this HIT. The experiment provides important information about the effectiveness of multidimensional visualisation techniques to convey data to a user. Any person observing and working with similar data may therefore benefit from more effective and meaningful visualisation.

Are there any possible risks from participating in this study?

There are no risks associated with this study. You are free to take as much time as needed and you can quit the experiment at any point without having to provide any particular reason

How will the data collected in this study be used?

The information that you provide for this research will be treated confidentially and will be used for the following purposes: CSIRO internal storage, statistical analysis and meta analysis with other experiments, research publications in conferences and journals. You will not be identified in any publications resulting from the study except where you have given written permission for this to occur. You may ask for part or all of the information provided to be removed from the study without penalty or explanation.

What if I have questions about this research?

If you would like to discuss any aspect of this study please contact Benedetta Frida Baldi (BenedettaFrida.Baldi@data61.csiro.au).This study has been approved by the CSIRO Human Research Ethics Board. If you have concerns or complaints about the conduct of this study you should contact the CSIRO Human Research Ethics Board +61 7 3833 5693 or csshrec@csiro.au.

Participant Consent

Please carefully review the information below:

- I agree to participate in the above project being conducted by CSIRO, Australia.
- I have been provided with information about the project and have any information about associated risks and benefits answered to my satisfaction. I understand my participation in the research will involve the following activities: viewing a three-dimensional model on a computer screen and judging which of the proposed two-dimensional plots encode for the same information.
- I have been informed that the duration of the experiment will be approximately 25-30 min.
- I have been provided with contact details of the investigating officers and understand that I can contact them at any point after the experiment for further inquiries. I have also been provided with the contact details of an independent ethics officer at CSIRO should I wish to raise any concerns or complaints about the conduct of the research.
- I understand that my participation in the experiment is entirely voluntary and that I am free to withdraw from the study at any time and without having to provide a reason for my withdrawal.
- I understand that I may ask for part or all of the information provided by me to be removed from the study without penalty or explanation.
- I understand that the information I provide for this research will be treated confidentially and will be used for the following purposes: CSIRO internal storage, statistical analysis and meta analysis with other experiments, research publications in conferences and journals.
- I will not be identified in any publications resulting from the study except where I have given my written permission for this to occur.
- I understand that according to the Australian Code for Responsible Conduct of Research and to CSIRO policy, the data will be retained for a minimum of 15 years, after which further need of the data will be reviewed. Any information provided by me will be stored securely by CSIRO and will not be accessible by any person outside the organisation.

Do you give your consent?

Yes No

Figure S1. Welcome page for the MTurk study. Show study description and participant consent form.

Explanation Section

As we mentioned, the first thing you will have to do is understanding a simple three-dimensional model. In particular you will have to determine which parts are in close proximity, but don't worry it's very simple. Lets start by looking at the 3D models that will be used in the study.

The three-dimensional model

The three dimensional model that you will see at the beginning of each question is a cylinder that has been segmented into 8 parts coloured in two shades of grey. We will refer to different segments by their number (1-8).
The cylinder has directionality meaning it has a distinct beginning and end. The model begins at **segment 1** which will be **light grey** and ends at **segment 8** which will be **dark grey**.

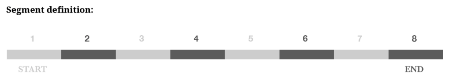

Spatial proximity definition

You will have then to establish which pair of segments are in close spatial proximity, meaning when they are close in space. We call this a **contact**. For each model only two segments can be in contact, and to make it very clear we coloured the segments that form a contact in **green**.
Segments in contact might be physically touching or not, it does not matter. When two segments are very close to each other and are **coloured in green this mean they form a contact**.

Contact definition:

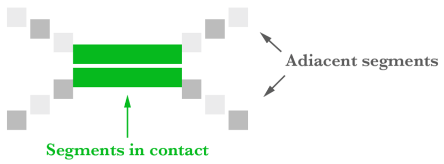

Example of a 3D model

As you can see in the image, the segments coloured in green, **segment 2** and **segment 5** are the ones that form the **contact**. Notice the direction of the model. The cylinder always starts with light grey and ends with dark grey independent from where it is in space - left or right, up or down.
So to recapitulate, **from these models you'll have to determine which segments form a contact**. In this case, segment 2 and segment 5.

3D example

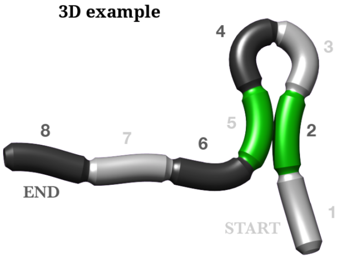

Next

Figure S2. Detailed explanation on how to read the three-dimensional model, for both experts and MTurk participants.

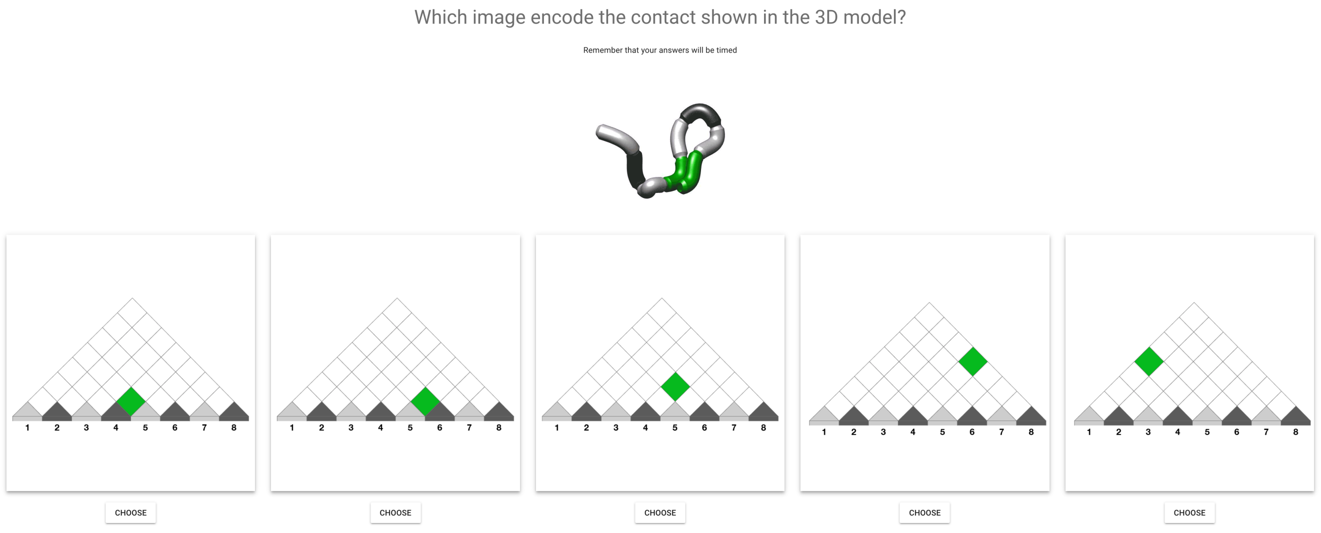

Figure S3. Example screenshot of one of the survey questions. Both experts and MTurk participants were asked to answer the same multiple choice questions
